# Racism in Europe: Characteristics and Intersections With Other Social Categories

**DOI:** 10.3389/fpsyg.2022.789661

**Published:** 2022-03-24

**Authors:** Elena Ball, Melanie C. Steffens, Claudia Niedlich

**Affiliations:** Social, Environmental, and Economic Psychology, University of Koblenz-Landau, Landau, Germany

**Keywords:** intersectionality, sexual orientation, class, gender, Europe, social identity, racism, labor market

## Abstract

Concerning race and its intertwinements with gender, sexual orientation, class, accents, or ability there is a scarcity of social psychological research in Europe. With an intersectional approach studying racism in Europe it is possible to detect specific experiences of discrimination. The prevalent understanding of European racism is connected to migration from the former colonies to the European metropoles and the post-Second-World-War immigration of ‘guest workers.’ Thus, the focus of this research is on work-related discrimination. Against the background of a short historical review, we present the results of the few existing studies on intersectional discrimination within the labor market in Europe and discuss their implications. The pattern of findings is more complex than the assumption that individuals belonging to two or more marginalized social categories are always the most discriminated ones. Gender, sexual orientation, and origin rather interact with the specific job context. These interactions determine whether minority individuals are discriminated against or even preferred over individuals belonging to the majority group. We argue that considering the stereotype content model and social-identity theory helps to structure the sometimes contradictory results of intersectionality research. Therefore, the review presents new perspectives on racism in Europe based on current research, develops hypotheses on the interplay of intersecting identities, and identifies four novel research questions based on racist attributions considering situational variables: These are the role of concrete job contexts in explaining (no) discrimination, the influence of different stereotypes regarding marginalized groups, the explanatory value of sexual orientation as well as class or socioeconomic-status and age in terms of some patterns of results.

## Introduction

And if I answer and say that I am German, they look confused, you know? They stop for a moment, like thinking: “German…?” Or they just start laughing, as if I misunderstood the question or gave the wrong answer, you know? And they go: “Oh! No, no! But you cannot be German. You don’t look German (*pointing to the skin*). Where are you from?” (Kilomba, 2008, p. 2).

In this situation, the person asking where the other one is from takes the power of defining what nationality one has. It implies that whether one is ‘German’ or not is defined by one’s skin color. The statement suggests the understanding of Germany as racially homogenous. Such views have grown differently in the historical development across nations. They still influence which stereotypes are present in people’s minds today so that in Germany a Black person is perceived as being a foreigner. In Europe, the category of foreignness which is often inferred from skin color and visible religious symbols is most relevant in defining belonging and distinction (Meer, 2013). Even though there are hints that also in Europe race has been and still is based on the visibility of phenotypic traits like skin color (Weichselbaumer, 2016), names, visible attributes like a head-scarf, a barb, or clothing, and even audible information like accents (Rakić et al., 2020) equally serve as markers that are used to identify people as non-White. Germany and other European countries outsourced a system of forced labor, societal segregation, and genocide to their colonies overseas (El-Tayeb, 2016). Although different European countries have slightly different tinges regarding their specific racialized groups and forms of racism, postcolonial immigration and the status of foreigners are at the center of the racial divide (Galonnier, 2015).

To summarize, in Europe, racism is based on a complex conglomerate of nationality, origin, accent, visual features, religion, and (ascribed) foreignness. An intersectional approach that is not built upon a “single-axis analysis” (Crenshaw, 1989, p. 139) is most suited for capturing this complexity of racism intertwined with other forms of discrimination such as that based on sexual orientation or socio-economic status (SES). We deem it important to focus on the hierarchically structured labor market as a highly relevant sphere since racism legitimizes such a hierarchical structure and secures economic profit and the maintenance of power and status differences (Balibar, 1991; Silverstein, 2005). Since money and opportunities for advancement are distributed here, changes in this area also entail consequences in other areas.

The aim of the present article is thus to delineate the facets of racism in Europe based on an intersectional stance. We acknowledge that research on racism in Europe has been conducted starting from a variety of theoretical approaches. Some of their tenets are more or less implicitly alluded to in the studies we are referring to: Cultural studies incorporate the concrete historic circumstances while analyzing racism in and through cultural phenomena focusing on popular culture and mass-media (Hall, 1999). Some proponents of social-identity suggest that it is productive to also look at the dominant group’s or ingroup’s self-conception instead of focusing on the question of whether individual prejudices are adequate or not (Reicher, 2007). Critical race theory (Delgado and Stefancic, 2001) and critical race psychology (Salter and Adams, 2013) shift their gaze away from prejudiced individuals and focus primarily on sociocultural contexts. We concentrate on theoretical elaborations based on intersectional approaches (Cole, 2009) within social psychology because our focus is on empirical evidence linked to intersectional racism.

The article is structured as follows: First, we define racism and present some historical facts characterizing racism in Europe to set the floor. Sometimes, we use the United States as the background in this article because most research on (anti-Black racism) was conducted there. Understanding the specific historical conditions makes different understandings of racism comprehensible. Second, we provide an overview of empirical findings of research on racism in Europe that used an intersectional approach to look at labor market discrimination. The latter findings are grouped against the background of their fit to four different fundamental frames on multiple minority membership or intersecting subordinate group memberships: The subordinate male target-hypothesis, the ethnic prominence hypothesis, the double jeopardy theory, and the theory of intersectional invisibility. We indicate the limitations of each approach with their partly contradictory findings. Third, we suggest that considering the cultural stereotypes of different groups and the insights from the well-established social-identity approach paves the way for reconciling those results. Such a multi-layered approach offers thus great potential for research in Europe doing justice to the complexity of racialized discrimination in interaction with other identities, not only making visible identities that have so far been largely invisible, but also uncovering in which contexts interconnections of identities entail disadvantages and when they do not.

## A Historical-Sociological Overview of the History and Functions of Race in Europe

### Definition of Race

To understand with what intersectional research on racism is dealing, we outline what the term ‘race’ encompasses and how it came about.

In general, the concept of racism operates through a hierarchical distinction between people. To achieve a deeper understanding of the concept, various political, social, legal, and educational practices have to be taken into account. From a sociological perspective, race can be described as a ‘fiction’ at first, but at the same time as the reality of various political and psychological issues: Races consist of their constitutive practices without an inner content (Mbembe, 2014). That is, races exist only because of the behavior that creates them. Population geneticists or researchers studying intelligence may find plausible arguments to group people into races but those groupings would not appear in the interpersonal realm if not looked for. Seemingly natural features are ascribed the meaning of essential racial characteristics (Silverstein, 2005). Overall, race can be understood as a flexible idea separating human beings based on a variety of characteristics, be it religion, culture, ancestry, ethnicity, or skin color, and be it named race, culture, background, or ethnicity (Roberts et al., 2020).

Dividing people into races was historically used in Europe in ‘racial science’ to create a hierarchy between races conceived as natural entities. This classification was strategically employed to justify colonial oppression and to build a hierarchically segmented labor market including enslaved workers (Balibar, 1991). The concept of race served thus to legitimize oppression, above all since the beginning of the 15th century when the trans-Atlantic slave trade began as one of the very brutal forms of oppression. It was “the first time in human history the principle of race and the subject of the same name were put to work under the sign of capital” (Mbembe, 2014, p. 33). The colonial conquerors were thus not only driven by the motive to seize foreign land but also to take part in an emerging capitalist economy. The core of this oppression was that human beings were racialized and devalued as a commodity ready to exploit. The economy included thus a mechanism to abuse human beings as unpaid workers to gain agricultural commodities and precious metals to meet the growing demand in Europe for such goods. In short, race and racism are historically grown ideas, respectively, practices serving capital-oriented goals in Europe and the Americas.

### Race as a Historically Shaped Hierarchy

As has been shown, hierarchies, as well as processes of downgrading and upgrading, play a specific role in understanding the function of race (Mbembe, 2014; Roberts and Rizzo, 2021). This means a hierarchy of different races with White people (men) at the top, supposed to embody the peak of human evolution. The ensuing dehumanization of specific groups of other people served to legitimize their – by the dominant group’s own standards – unfair and cruel treatment (Bonilla-Silva, 2007). Thereby, the dominant group maintained a system of colonialist slavery, forced labor, and, thus, economic profit securing power and status differences. The constructed racial hierarchy created both symbolic and material differences between the oppressed and the oppressor. These differences nowadays erroneously appear natural, normal, and legitimate, and they were upheld to not contaminate the purity of the so-called ‘superior’ White race. Following Gillborn (2016) these processes are today still at work in the United States and Europe as denoted by the term ‘White supremacy.’

Until the Civil Rights Movement, the institutional anchoring of racist practices was legitimized by different laws in the United States based on legal institutions dating back to the time of slavery (Delgado and Stefancic, 2001). In contrast, in Germany, there was no comparable legal anchoring, except for the time of National Socialism and, to a lesser extent, the legislation concerning ‘guest workers.’ Under National Socialism from 1933 until 1945, Germany was meant to be purged of so-called foreign races, including Jewish people, Rom:nja and Sinti:zze, and Black people to establish a purely Aryan nation (Fings, 2015). These ideas did not just disappear from people’s minds after the time of National Socialism in Germany and its partly allied regimes in other European countries: Occupation-children born to White German mothers and Black GI-soldiers after World War II were seen as a threat to the German nation and their release for adoption by Black Americans was discussed in the German parliament (El-Tayeb, 2016). The stigmatization and discrimination of Rom:nja and Sinti:zze in official (police) statistics (Widmann, 2015) persisted just like Antisemitic attacks (Antidiskriminierungsstelle des Bundes, 2021). The end of World War II was thought of as a caesura, establishing a Germany without antisemitism, right-wing nationalism, and racism (El-Tayeb, 2016). Yet, ideas of ‘Volkstum’ based on descendance and consanguinity were kept alive (Arndt, 2017) and were even strengthened when people who were seen as fundamentally and essentially ‘other’ entered the country as migrant workers or gained visibility (Foroutan, 2013). But generally, in Europe, race was externalized and put to work in the colonies (also creating tribes imagined as distinctive entities Lentz, 1995) as the enslaved African people were mostly not brought to the European continent for forced labor (Lentin, 2008). This short historical overview sketches the specificities to the construction of race in Europe.

### Linkage Between Racism and Migration in European Countries

For interpreting research on racism, the fact that European countries do not see themselves as immigration countries is important. Even though there are noticeable differences between European countries regarding the national self-concept and immigration policies they share that they do not see themselves as immigration countries and that, as former colonial countries, immigration increased in the 20th century.

In Germany, racism is mainly discussed in the context of foreigners and the migration of ‘guest workers’ from other European countries or former colonies (Balibar, 1991; Silverstein, 2005). In contrast, in France, immigration from its former colonies is discussed from the perspective of socio-economic integration and inclusion in French universalism (Silverstein, 2005), whereas in the United Kingdom, the debate centers around race relations alluding to a postcolonial situation (Balibar, 1991). France and Great Britain grant citizenship to people who are born on their territory and to people whose parents are already citizens of the respective country. In Germany, the principle of descent is still prevailing, and having two nationalities is possible only with a few other countries.

During the colonial period, people who were seen as foreigners, coming from the colonies lived and worked in the colonizing nations. Still, they migrated in relatively small numbers to the ‘motherlands.’ The first time that foreigners migrated in higher numbers to Germany was when people from adjacent countries were hired as so-called ‘guest workers’ with the first recruitment agreement in 1955 (Bundeszentrale für politische Bildung, 2010). Even though the legal framework was intended to secure their rotation and impede their settlement in Germany, some of them stayed and built a life for themselves and their families. This was the case despite physically demanding and dangerous work in the lower wage range and an arbitrary allocation and revocation of work permits. So, in spite of an enhanced promotion of return and a recruitment stop since the 1970s, Germany has become a country of immigration (Butterwegge, 2005). After the collapse of the Soviet Union and the civil war in Yugoslavia, there was a second wave of immigration toward Germany. It was the first major movement of refugees after World War II. Since the so-called Arab Spring and its ensuing civil wars, another influx of refuge-seeking people took place from 2015 onward (Oltmer, 2016). The diversity within Germany has increased since then, but so have, once again, racist, right-wing extremist, and anti-Semitic crimes (with the exact numbers depending on the counting method, because in Germany, racist incidents are not recorded uniformly and systematically (Oltmer, 2016; Brausam, 2021). People are still treated differently because they belong (allegedly) to social groups viewed as different from the norm as can be seen in the significant increase for counseling on experienced discrimination in 2020 in Germany (Antidiskriminierungsstelle des Bundes, 2021).

In sum, the above historical outline with a focus on the German realities has shown the intertwining of racism, migration, and capitalist economic development. Racist ideas served as a legitimizing ideology for exploitation to procure cheap labor for low-skilled work.

At present, the situation has somewhat changed compared to the beginnings of migration movements: The offspring of the ‘guest workers’ in Germany achieves generally a better education than their parents despite racism in educational establishments (Schu, 2020). On a European level, over 30,000 highly skilled immigrants received a blue card in 2019 to work within the European Union (Eurostat, 2020). Therefore, we deem it important to look at forms of racist discrimination intersecting with other forms of group-based discrimination in the work context in Europe, considering that both negative, primarily group-based perception and more positive, individualized perception of successful people should occur in parallel.

### The Silence About Race in Europe

That being White constitutes the norm is another connecting feature between different European countries, where the legacies of the European colonial period are not as actively discussed as, for example, the legacies of slavery in the United States. Due to historic parallels and “as a consequence of both the reluctance of many European nation-states to deal with their colonial history and the widespread notion that Europe indeed consists of many different ethnicities, who, however, all belong to the same ‘white race”’ (Wandert et al., 2009, p. 5) similarities exist between various European countries: An unnamed whiteness was set as the norm in the process of the construction of Europe (Mbembe, 2014; Arndt, 2017). The psychological mechanisms behind this can be illuminated by research on asymmetric explanations for group differences: Higher-status groups are the ones that are perceived as being more prototypical than lower-status groups so that lower-status groups are the ones that are differentiated, named, and labeled as the deviation from the norm (Hegarty and Bruckmüller, 2013). White people forming the high-status group are thus the background against which non-White people are perceived as diverging. Since no category for the analysis of racist experiences exists due to the deletion of the notion of race, De Genova (2018) speaks of postcolonial amnesia in Europe: “Banishing race as a critical analytical category, in other words, risks forsaking any adequate account of the distinctly European colonial legacies that literally produced race as a sociopolitical category of distinction and discrimination in the first place” (De Genova, 2018, p. 18). Thus, instead of taking race as an empirically given, scientific truth, it can be seen as a political reality as described above when talking about races as sociopolitical positions lashed in racism. Only then, it is possible to analyze the important role of racism in a post-colonial world.

The reluctant recognition of the existence of racism is based on the silence about race and reflected in a silence about what it means to be White. Even if the silence about whiteness seems to be most pronounced in Germany, Arndt (2017, p. 24) points out that not only Germany, but the whole of “Europe is not a religiously and culturally homogeneous ‘natural’ entity, but rather a historical and political construct, which sought to give itself form and content above all in its external, especially in its demarcation from the outside.” Similarly, what was then the German Empire was based on a multitude of diverse peoples with large cultural and linguistic differences. Demarcating a German nationality based on being White and Christian was also meant to offer a common identity in the nation-building process (Arndt, 2017). Likewise, the construction of a European identity through a distinction based on Roman law, Christianity, and the epoch of Enlightenment is intricately linked to the category of whiteness (Arndt, 2017).

Thus, European racism is a specific configuration of institutional phenomena, linked to the formation of Europe and a European self-image that De Genova (2018, p. 14) describes as a “racial formation of postcolonial whiteness”. In racialized European societies being White means conforming to the norm and thus being perceived as truly European.

## Different Forms of Racism Within Europe

Whereas being White is constructed as the European norm, different groups of people based on varying features are seen as non-White. The differential racialization thesis stressed by critical race theory accounts for the fact that each racialized group “has been racialized in its own individual way and according to the needs of the majority group at particular times in its history” (Delgado and Stefancic, 2001, p. 69). In Germany, this can be exemplified in stereotypes and discrimination of Rom:nja and Sinti:zze based on demonizing-eroticizing ideas linked to musicality, permissiveness, and crime, while Muslims are constructed and discriminated as being the counterpart to the ‘West,’ lacking secularism and women’s rights, and being associated with a cultural threat or terror attacks (Arndt, 2017). This association is reflected in corresponding stereotypes (Rexhepi, 2018; Fröhlich and Schulte, 2019).

The distinction between different forms of racism (i.e., against Rom:nja and Sinti:zze, anti-Muslim racism, anti-Asian, antisemitism) should not lead to establishing a hierarchy of victims (Delgado and Stefancic, 2001, p. 69). But in order to fully understand the experiences individual people make, it is helpful to describe the specific forms of oppression they encounter based on the respective stereotype content associated with them. This is what the framework of intersectionality aims to show: That the intersections of social identities connected to race, gender, class, dis/ability, or age produce different advantages and disadvantages for the people affected (Purdie-Vaughns and Eibach, 2008).

One form of racism, anti-Muslim racism, has been extensively researched in Europe compared to other racisms. For example, in a survey from 2011, Zick and colleagues interviewed representative samples of inhabitants of eight European countries to assess their group-focused enmity. The conclusion was that “Europeans are largely united in their rejection of Muslims and Islam” (Zick et al., 2011, p. 63). Corresponding results can be seen in the European Islamophobia Report (Bayrakli and Hafez, 2019) which underlines that Muslims are seen as a new racialized faith group distinguished by characteristics like hair, beard, clothing, and sometimes skin color (Meer, 2013).

Because detailed elaborations on this much-researched topic exist – see for example Foner (2015), Galonnier (2015), Sharma and Nijjar (2018) – in the following we will focus on other forms of racism as well. Still, intersectional research on anti-Muslim racism is rather rare and that’s why we included empirical intersectional research on this specific form of racism. European countries not only have various links to Islam and a Muslim population (El-Tayeb, 2016), they also comprise Black populations established since the colonial period. Their number increased especially during World War I and II when men from the respective colonies were recruited to serve in ‘colonial troops’ (Fischer-Tiné, 2016). Further, European countries have only little experience with affirmative action naming and tackling racist discrimination (Imoagene, 2012). This could be one reason why it is easier to deny the existence of racism in Europe compared to the United States, and why there is a focus on discrimination and violence against foreigners instead of a part of one’s own population. With European countries and, especially, Germany having a tradition of often not broaching the subject of racism with reference to their differing history from the United States (Alexopoulou, 2018) it is important to research forms of discrimination and mechanisms of oppression in Europe specifically.

As Di Stasio and Larsen (2020, p. 246) note, “studies conducted outside of the United States context are needed to assess existing findings on gendered racism in a comparative perspective.” The idea of gendered racism suggests that different races are associated with differing masculinity or femininity such that Black people are seen as more masculine than White people and Asian people as rather feminine compared to White people (Galinsky et al., 2013; Hall et al., 2015). Even more generally, we posit that an intersectional perspective on discrimination based on race intertwined with gender, age, or class within European countries is needed to integrate the few existing research strands.

## Empirical Pieces of the Puzzle

Intersectional social psychological research on workplace discrimination in Germany has raised more questions than answers so far, as we want to illustrate in the following section. At the same time, different findings contain some hints that pursuing these questions further offers insights into the mechanisms of racist discrimination in Germany and other European countries.

Two recent studies we conducted, described in the following, showed that the stereotype content for Black people in Germany does not mirror exactly that from the United States context. We focused on warmth (or communion) and competence (or agency), described as the two fundamental dimensions of person perception by the stereotype content model (Fiske et al., 2002). The combination of high/low communion and high/low agency produces mixed stereotypes for specific groups combined with distinct emotions. For example, a paternalistic stereotype of Black people portrays them as low in competence and high in warmth needing help, deserving a subordinate position and evoking pity or contempt. In contrast, an envious stereotype sees lesbians as high in competence but low in warmth posing a potential threat and evoking feelings like envy or jealousy, while traditional women like housewives are rather met with paternalistic stereotypes receiving pity and sympathy (Fiske et al., 2002). The two-dimensional stereotype content model can thus help to explain from a social psychological perspective why a differential racialization (Delgado and Stefancic, 2001) as described above happens: because different stereotypes are applied to groups racialized to varying degrees and with different nuances.

The two fundamental dimensions of competence or agency and communion or warmth relate also to consolidating social connections and striving for goals, respectively (Abele and Bruckmüller, 2011). Each can be divided further into two facets: Assertiveness and competence are facets of agency; morality and warmth are facets of communion (Abele et al., 2016).

All of these facets (also) play a central role in work contexts. In a preregistered survey on cultural stereotypes about Black men and women in Germany described as either heterosexual or gay/lesbian, we found that a shared stereotype was their assumed foreignness (Ball, unpublished^1^). This survey was based on a list of stereotypes that included stereotypes about Black and White heterosexual men and women gauged in the United States (Ghavami and Peplau, 2012) and stereotypes collected in a free-response format in Germany regarding Black heterosexual and gay/lesbian people and, as a baseline condition, White heterosexual men and women. The collection of cultural stereotypes used can be found in Table 1. Participants (*N* = 24) were asked to name at least three and up to ten stereotypes that they deemed as most important describing those social groups. The responses generated through this free-response format were analyzed by a qualitative content analysis allowing to reduce the data through combining synonyms and inducing umbrella categories (Mayring, 1994). Participants had named 84 respectively 81 stereotypes for Black heterosexual women and men, while for Black lesbians and Black gay men 65 respectively 66 stereotypes were named. Two coders selected the stereotypes from the list presented by Ghavami and Peplau (2012) that appeared appropriate in the German context, relevant for the working context, or connected with agency and communion (e.g., “like to eat fried chicken” was thus not adopted) and independently translated them into German. Any disagreements among the coders were discussed until they reached a consensus.

**TABLE 1:** List of stereotypes presented (Ball, unpublished).

Intelligent *(intelligent)*	*Assertive (durchsetzungsfähig)*
*Arrogant (arrogant)*	Aggressive (aggressiv)
*Erfolgreich (successful)*	Sexually attractive (sexuell attraktiv)
Domestic (familiär)	*Lazy (faul)*
Foreign (fremd)	*Violent (gewaltbereit)*
Lack of German language skills (mangelnde Deutschkenntnisse)	Emancipated (emanzipiert)
Ungebildet (uneducated)	Masculine (maskulin)
Friendly (freundlich)	Refugee (geflüchtet)
Feminine (feminin)	Strong (stark)
Liberty-seeking (freiheitssuchend)	*High status (hoher sozialer Status)*
Conformist (angepasst)	Leadership skills (*Führungsstärke)*
Extroverted (extrovertiert)	Arrogant (arrogant)
Feisty (temperamentvoll)	*Ditsy (schusselig)*
Loud (laut)	*Racist (rassistisch)*
Dominant (dominant)	*Emotional (emotional)*
Have an attitude (störrisch)	*Submissive (unterwürfig)*
*Confident (selbstsicher)*	*Sexist (sexistisch)*

*Adjectives derived from Ghavami and Peplau (2012), are printed in italics, the German translation used is given in parenthesis.*

In the ensuing survey participants (*N* = 43) were asked to indicate their agreement with each of these 38 stereotypes based on the 10 most frequently mentioned ones from the first survey and the list from Ghavami and Peplau (2012) toward the social groups in question. As can be seen in Table 2 the specific German stereotypes were endorsed more than the American ones. Further, the assumed foreignness is the most marked contrast compared to the other stereotypes ascribed to Black people both in Germany and the United States, which showed some similarities, especially regarding physical features. White German participants ascribed Black heterosexual men an intense, sexualized masculinity, while Black heterosexual women’s sexual availability was emphasized along with their dominant, loud behavior. Black gay men were attributed a stigmatized gay identity and Black lesbians were described as invisible yet liberated by the participants.

**TABLE 2:** Agreement with American and German stereotypes.

Stereotypes	American stereotypes		German stereotypes	
	*M*	*SD*	*M*	*SD*
Black heterosexual women	3.70	0.40	4.16	0.33
Black heterosexual men	3.78	0.50	4.49	0.54
Black lesbian women	3.63	0.55	4.20	0.32
Black gay men	3.60	0.37	4.17	0.32
White heterosexual women	4.02	0.44	3.63	0.51
White heterosexual men	4.27	0.49	3.23	1.01

We compared those results with the results of another experiment from our lab (Bohlender, 2016) which examined stereotypes of people of Turkish origin in Germany: Here, Turkish heterosexual men were described by participants (*N* = 72) as performing a stereotypical, dominant idea of masculinity, whereas German heterosexual men were described as rather competent. It thus appears that the two facets of agency, dominance and competence, were split between the two groups: German heterosexual men were seen as rather competent, whereas Black and Turkish heterosexual men were seen as rather dominant. In contrast to that, Black gay men and Turkish gay men were both seen as predominantly gay and were no longer described as prototypical of their racial group. Taken together, there are some similarities in the stereotypical perception of Black heterosexual women and men in Germany compared with the United States regarding the ascribed dominance, less competence, and a sexualized view. But we notice also a striking difference even extending to Black lesbians/gays concerning their perception as being foreigners or refugees. This should be due to the historical specificities in Europe described above. In exploring this further we aim to build on the existing research on discrimination in Europe based mainly on race, gender, and sexual orientation.

### Intersectional Complexity: Some Theoretical Highlights

Before discussing the research on intersectionality within Europe some further theoretical background is needed. Gendered job contexts interact with applicants’ group memberships, mainly with their gender. Technical and scientific jobs are predominantly carried out by men. According to social role theory (Eagly and Wood, 2012), gender stereotypes arise when we observe women and men showing different behavior, thus enacting different social roles. Inferred from these different social roles are the above-described dimensions of person perception of agency and communion that are linked primarily with women and men, respectively (Diekman and Eagly, 2000). As these two traits are also inferred from a person’s professional role a gender-segregated job market leads to differing ascriptions of communion and agency. From the differing behavior connected with their jobs, it is then conferred that men and women possess different qualities and traits. Just as is the case with the idea of races and their confirmation in racism, imagined gender differences are affirmed through sexist practice. Lower qualified or blue-collar jobs in the technical-craft sector are predominantly done by men and thus activate more gender stereotypes leading to increased masculinity of job contexts. Thereby discrimination arises especially toward women who apply for such jobs (see Güngör and Biernat, 2008). High-status jobs, especially leadership positions, are also generally seen as requiring dominant, typically masculine behavior. Thus, women applying for such jobs are considered to be less suitable (Heilman, 1983; Eagly and Karau, 2002), unless these women have other characteristics besides their gender that are stereotypically associated with dominance, such as being Black (Rosette et al., 2016) or being lesbian (Niedlich et al., 2015).

As far as the importance of the qualification level of a job is concerned, findings are mixed, with field experiments detecting both more discrimination for lower educated men and women from different minority groups in the Netherlands (Andriessen et al., 2012) and Belgium (Derous et al., 2012; Derous and Pepermans, 2019) and no discrimination for Arab women applying for high- and low-status jobs in the Netherlands (Derous et al., 2015). Especially strong discrimination of Latina women (but not Latino men) applying for higher-skilled (and not typically female) jobs was found in Spain (Yemane and Fernández-Reino, 2019). Likewise, in Belgium, Arab women applying for high-status jobs were discriminated against (Derous and Pepermans, 2019). Similarly, a study in Germany (Weichselbaumer, 2020) found that Muslim women wearing a headscarf and applying for high-status positions experienced more discrimination than when applying for lower-skilled jobs. As an explanation for the last result, the author proposes in line with the symbolic threat argument that the highly visible feature of a headscarf is seen as quite inappropriate for a high-status position. Furthermore, employers may make more conservative hiring decisions when choosing people for jobs of which there are fewer and which require higher qualifications. With regard to the state of the labor market, it is impossible up to now to conclude whether a higher number of vacancies increases job-related discrimination or vice versa as there are study results supporting each assumption (Baert et al., 2013, 2015; Blommaert et al., 2014; Carlsson et al., 2018).

Given those divergent findings, we identify as Research Need 1 that future studies on workplace discrimination should consider the status of the job and the qualification level needed. The results described in this section with some findings depending on target gender imply that using an intersectional approach should reveal further differences. We, therefore, present in the following different predictions regarding intersectional targets along with corresponding empirical evidence. The section is organized around the four most influential theories that consider the interplay of multiple marginalized social identities: The subordinate male target hypothesis focuses on the role of male members of marginalized groups; double jeopardy assumes an addition of racial and sexist discrimination for non-White women; the ethnic prominence hypothesis emphasizes the primacy of racialized group membership over other group memberships; finally, the theory of intersectional invisibility posits that belonging to multiple marginalized groups can be associated with advantages or with disadvantages, depending on specific group memberships and stereotype content.

### Social Psychological Research on Intersectionality in Europe

A central tenet of all intersectional approaches is the idea of a “rubric of intersectionality, which feminist and critical race theorists developed to describe analytic approaches that consider the meaning and consequences of multiple categories of social group membership” (Cole, 2009, p. 170). The focus is put on the various ways that multiple group memberships define people’s asymmetric relations to one another. This, in turn, influences people’s opportunities, experiences, and evaluations by others. Such an intersectional perspective allows grasping the complex reality of social category membership, dominance, and oppression and how they are negotiated. Yet, there are only few empirical studies looking through an intersectional lens at job-related discrimination in Europe. In the following, we illuminate those research results in light of four main theories all of which attempt to do justice to the phenomenon of intersectionality grasping it in different ways. We are not going to detail other theories mentioned in the studies in order to reduce complexity. We discuss the respective specificities the four theories take into account as well as the empirical evidence obtained.

### Subordinate Male Target Hypothesis

The first approach, the subordinate male target hypothesis (SMTH) is derived from social dominance theory (Sidanius et al., 2000; Sidanius and Pratto, 2001). Based on evolutionary reasoning, it suggests that outgroup men are the main target of racism. Whereas the construction of the categories of women and men as the basis of a patriarchal order differs only slightly across times and places, the relevant group distinctions for other forms of inequality are highly variable. They range from differentiation based on race, class, and nationality to language and are thus described as arbitrarily chosen (Sidanius et al., 2000). Due to intrasexual competition among men for reproductive access to women, social dominance theory predicts “aggressive male coalitions to establish expropriative and hierarchical relations against the resistance of outgroup males” (Sidanius et al., 2000, p. 12). One would thus expect that men belonging to a racial minority are more discriminated against than women belonging to the same racialized group.

Some studies found evidence for this hypothesis (for an overview, see Table 3). An experiment in the Netherlands showed that in gender-neutral jobs with both high customer contact and high/low cognitive demands discrimination against Arab men was more pronounced than against Arab women (Derous et al., 2015). This effect was more pronounced when a higher number of ethnic cues (e.g., being a member of an Arab association as well as having an Arab name) made this social category especially salient. More evidence for the SMTH was provided by another study conducted in the Netherlands (Andriessen et al., 2012) that used not only written applications with different names and birthplaces but also actors with different names and accents for in-person applications. Turkey, Morocco, the Antilleans, and Surinam were chosen as countries of origin to compare immigrant groups with different statuses. Overall, discrimination against those with a migration history was found across the different migrant groups independent of the ethnic hierarchy. In terms of interactions of gender and ethnicity, the authors found weak evidence for the SMTH: Ethnic minority women were slightly more likely than ethnic minority men to be invited for job interviews compared to native Dutch men and women. In jobs that required a lower level of qualification, there was slightly more discrimination than in intermediate and high skilled jobs. Discrimination was slightly lower for jobs with little customer contact for all immigrant groups. Another piece of evidence for the SMTH was found in Denmark (Dahl and Krog, 2018). Experimental conditions were gender (male/female), crossed with ethnicity (Danish/Middle-Eastern names), and with SES (high/low SES for the Danish applicants). Minority men with Middle-Eastern sounding names received fewer callbacks than minority women for their applications both in female- and male-typed and gender-neutral jobs requiring intermediate levels of qualification. In work contexts where the share of women was under 20%, this discrimination was most pronounced. A final piece of evidence for the SMTH comes from a field experiment using comparable CVs for real job advertisements in Sweden (Arai et al., 2015) that manipulated the amount of job experience applicants with Arab names had. Both Arab men and women with equal qualifications like the Swedish applicants experienced discrimination when they applied for male- and female-typed jobs of high and intermediate skill levels. Arab women needed to indicate having one to three years more experience than their Swedish counterparts to no longer experience discrimination. Thus, the Arab women had to be better qualified (with possibly a worse starting position so that they had to perform better than members of the majority group) to get the same job as the Swedish applicants. This finding comes with one exception: When Arab women applied as drivers, they were still at a disadvantage compared to Swedish men and women. The Arab men, though, were still discriminated against no matter how many years of additional job experience they had. Arai et al. (2015) propose that the different findings regarding Arab women and men are due to the counter-stereotypic behavior of the Arab women who disconfirmed the group stereotype of domestic housewives simply by being active in the labor market. But then, this effect should be found also when Arab women applied for the typically male job as drivers, which was not the case.

**TABLE 3:** Overview of European social psychological studies on labor market discrimination based on the subordinate male target hypothesis.

Study	Sample	Theoretical framework	Job context	Social identities considered	Results
Andriessen et al., 2012 Netherlands	Correspondence test: 1080 paired applications	Multiple group membership based on gender and ethnicity, taste-based vs. statistical discrimination, ethnic hierarchy based on cultural distance	Low/high amount of customer contact, low/intermediate/high skill level: finance, local government, retail, hospitality industry, health care	Men/women of Turkish, Moroccan, Antillean, Surinamese ancestry/Dutch people	Discrimination_*ethnic minority members*_ > discrimination_*Dutch applicants*_, especially for low skilled jobs and with more customer contact; discrimination_*ethnic minority men*_ > discrimination_*ethnic minority women*_
Arai et al., 2015 Sweden	Correspondence test: experiment 1: 566 paired applications, experiment 2: 584 paired applications	Taste-based and statistical discrimination	Intermediate/high skill level, female-/male-typed: computer specialists, high-school teachers, nurses, economists, drivers	Swedish/Arab men/women	Discrimination against Arab men and women when they applied for male- and female-typed jobs of high and intermediate skill level; no longer discrimination against Arab women with three more years of experience than their Swedish counterparts (except for drivers)
Dahl and Krog, 2018 Denmark	Correspondence test: 800 paired applications	Taste-based and statistical discrimination, intersection of gender and ethnicity	Female- and male-typed jobs and gender-neutral jobs, intermediate skill level	Men/women with Middle-Eastern/Dutch names	Discrimination_*minority men*_ > discrimination_*minority women*_ in all areas, especially in female-dominated jobs; discrimination_*Middle Eastern applicant*_ > discrimination_*Danish applicant*_ if the latter had a name signaling a low SES
Derous et al., 2015 Netherlands	Experiment: 60 Dutch recruiters, 124 Dutch recruiters^1^	Multiple category membership, job context, rater differences and ethnic salience as potential moderators attributional ambiguity	Low/high amount of customer contact, gender-neutral jobs, low/high cognitive demand	Dutch/Arab men/women with differing amount of ethnic cues due to affiliation with an Arab/Dutch organization (all Arabic, all Dutch, mixed affiliation and ethnicity)	Discrimination_*Arab women*_ < discrimination_*Arab men*_ when applying for high and low cognitive demand jobs with much client contact; discrimination against Arab persons compared to Dutch persons when applying for jobs with high and low amount of client contact

*^1^Rater characteristics: implicit/explicit prejudice (IAT/Modern Racism Scale), implicit/explicit sexism (IAT/Modern Sexism Scale).*

In spite of the solid evidence base for social dominance theory in general and all the real-life examples that Sidanius et al. (2000) brought forward for his hypothesis, we consider the evidence from respective studies on the SMTH in Europe as weak. Taken together, some evidence from the Netherlands (Andriessen et al., 2012; Derous et al., 2015), Denmark (Dahl and Krog, 2018), and Sweden (Arai et al., 2015) can be interpreted as supporting the SMTH. A study comparing the SMTH against two other theoretical ideas (see below: ethnic prominence hypothesis and multiple minority status) in the Netherlands for both high- and low-qualified jobs (Derous et al., 2012) did not find evidence in support of the SMTH. The study by Andriessen et al. (2012) showed primarily discrimination against members of racialized minorities, irrespective of their gender. And in Arai et al.’s (2015) study women experienced discrimination just like men except when they had more work experience and when not applying as drivers. Thus, out of five experiments that could have served as a test of the idea of subordinate men as the main target of racial discrimination, only two yielded clearly the hypothesized pattern of findings.

Other researchers stress that it is ethnicity with its visible markers that is the most relevant social category. This implies that people of all genders belonging to a marginalized ethnic group are discriminated against compared to people from the dominant White group. What is the evidence for this hypothesis?

### Ethnic Prominence Hypothesis

The ethnic prominence hypothesis (Levin et al., 2002) holds that ethnicity is a more prominent social category than gender because marginalized ethnic groups are typically a minority compared with women actually outnumbering men in many countries (Statistisches Bundesamt: Destatis, 2021). Moreover, discrimination based on ethnicity or race is generally more conflictual and present in the public domain than that based on gender (sexualized violence, for example, often occurs in the private sphere). Two experiments, one in the Netherlands (Derous and Ryan, 2012) and the other one in Belgium (Derous and Pepermans, 2019), found evidence for both the ethnic prominence hypothesis and double jeopardy as an alternative explanation (discussed in the next section).

In the Netherlands, Derous and Ryan (2012) showed that Arab men were discriminated against more than Arab women when applying for gender-neutral jobs requiring intermediate qualifications, but only if both the men and women indicated activism in a Dutch and not an Arab Youth association. In fact, Arab women being active in an Arab Youth association and thus highly identified as Arab were rejected more often than their male counterparts. Even though this finding was not significant the authors interpret it as supportive evidence for the prominence of ethnicity when an individual is highly ethnically identified so that gender differences are being overlaid. A similar study in Belgium (Derous and Pepermans, 2019) showed that people with a Moroccan background were discriminated more than Belgians in low-skilled jobs, lending support to the ethnic prominence hypothesis.

When manipulating applicants’ skin color another study in Belgium (Derous et al., 2016) found evidence for the ethnic prominence hypothesis. Experimental conditions were the applicant’s name (Arab/Flemish), crossed with a light versus dark skin tone, and with the job context (high or low client contact and high or low industry status). Equally qualified male applicants with dark skin color received lower job-suitability scores by a sample of recruiters than applicants with light skin color. This was especially the case when the pool of applicants was screened for high customer contact/low industry status positions and low customer contact/high industry status positions. Another ethnic cue, an Arab-sounding name, was not connected to lower job-suitability ratings. The authors propose that in high-status jobs with less client contact applicants with a dark skin tone may have experienced more discrimination because their advancement there was seen as a more intense threat to majority group members. Since only men were studied as applicants it still needs to be tested whether these findings apply to women as well and can thus be interpreted solidly from the perspective of the ethnic prominence hypothesis.

In Austria, a field experiment (Weichselbaumer, 2016) compared waiting time and callback rates for women and men with and without a migrant background. Names and photos were used to signal applicants’ origins. Two districts of Vienna with comparable socio-economic structures were used as the return addresses, thus ruling out class-differences as the basis for discrimination. The experiment revealed substantial discrimination against applicants with a Serbian, Turkish, Chinese, or Nigerian background. The strongest discrimination was found against people of African origin, independent of the required team/customer contact in different positions. Similar effects were found in Germany, the Netherlands, Norway, Spain, and the United Kingdom (Veit and Thijsen, 2019): Experimental conditions in this international field experiment were place of birth (domestic-/foreign-born) and ancestry (European/African, Middle-Eastern). Foreign-born applicants of African or Middle-Eastern origin were discriminated against more than applicants who originated from other European countries. When applying for a variety of male- and female-typed and both academic and non-academic job offers candidates with an African or Middle-Eastern background received considerably less callbacks than domestic-born applicants or applicants with origins in another European country. Still, when analyzing the results for each country separately, the authors (Veit and Thijsen, 2019) stress that there are important variations regarding the combined effect of birthplace and ancestry such that, for example, in Spain being born in an African/Middle-Eastern country is connected with higher discrimination than in Germany. That’s why Veit and Thijsen (2019) emphasize how important it is to consider the entire socio-political context of national history, legislation, and anti-discrimination policies. Their findings relate to a study in Finland (Liebkind et al., 2016) which explicitly took ethnic hierarchies between people regarded as belonging to an out-group in Europe into account. Experimental conditions here were country of origin (Finland/Austria/Poland) in their first study and country of origin (Russia/Finland), gender (male/female), job type (typically male/female) in their second study. When comparing the results for male dentists applying for a position in Finland and either originating from Austria (higher-status minority group) or Poland (lower-status minority group) the Austrian immigrants were preferred over the Polish ones. The Austrian applicants were even as likely as the native Finnish ones to be chosen for the job opening. Thus, the ethnic prominence hypothesis with the restriction of it applying only to a low-status minority group received support. Besides that, we argue that the study results underline the multi-faceted idea of race within Europe: Visual or acoustic cues are interconnected with nationality as all the applicants were considered to be White apart from their differing national origins. In the second part of the study (Liebkind et al., 2016), when migrants from Russia applied for semi-skilled jobs which were either typically female or male, Russian men and women were discriminated against compared to Finns. Here, again, the ethnic prominence hypothesis for lower-status immigrants was supported. When Russian men applied for typically female jobs, they were discriminated against more than Russian women applying for typically male jobs. The authors do not interpret this finding as evidence for the SMTH we discussed in the previous section, but rather point toward specific negative threat-connected stereotypes of Russian men in Finland due to historic experiences of military aggression (Liebkind et al., 2016). Altogether, all cited studies (see Table 4) show some evidence for the ethnic prominence hypothesis pointing toward the importance of the ascribed ethnicity in discriminatory behavior. Yet, one weakness of the studies by Derous et al. (2016) and Liebkind et al. (2016) is that they (partly) only looked at male applicants. The conclusion that the results support the ethnic prominence hypothesis is therefore not yet entirely convincing. Moreover, there seems to be a fairly complex interplay with other factors, so there are different boundary conditions that need to be considered: The gender-typicality of the job applied for, its status, and the qualifications required along with customer contact and the hierarchy among different ethnicities influence who experiences discrimination compared to another minority group or the majority group. Two studies presented also some evidence that minority ethnic women are especially discriminated against in some situations (Derous and Ryan, 2012; Derous and Pepermans, 2019)2. This will be further explored in the next section drawing on theoretical reasonings focusing on women and long-established gender hierarchies besides a racial rule. They emphasize that women of different races, class statuses, and sexual orientations are always subject to sexism, too.

**TABLE 4:** Overview of European social psychological studies on labor market discrimination based on the ethnic prominence hypothesis.

Study	Sample	Theoretical framework	Job context	Social identities considered	Results
Derous and Ryan, 2012 Netherlands	Correspondence test: 600 paired (4 résumés each) applications	Ethnic prominence hypothesis, subordinate male target hypothesis	Intermediate skill level, gender-neutral jobs	Women/men with Arab names with activism in Dutch/Arab Youth organization	Discrimination_*Arab men*_ > discrimination_*Arab women*_ when applying for gender-neutral jobs requiring intermediate qualifications but only if they indicated activism in a Dutch and not an Arab Youth association; rejection_*Arab women*_ > rejection_*Arab men*_ when both highly identified as Arab
Derous et al., 2016 Belgium	Experiment with 424 Belgian recruiters	Colorism, ethnic prominence-hypothesis	Low/high amount of client contact, low/high industry status	Arab men with dark/light skin tone	Discrimination_*applicants with dark skin color*_ > discrimination_*applicants with light skin color*_ for high customer contact/low industry status positions and for low customer contact/high industry status positions
Derous and Pepermans, 2019 Belgium	Experiment: 214 Belgian recruiters	Double jeopardy hypothesis, ethnic-prominence-hypothesis	Low/high cognitive demand (clerk/manager)	Maghrebian/Arab men/women, high/low socioeconomic status of the Dutch applicant	No discrimination against Maghrebian/Arab women applying for low-cognitive demanding jobs in Belgium; when not considering job-context there was no difference in discrimination against Maghrebian/Arab men and women
Liebkind et al., 2016 Finland	Experiment: 103 students as recruiters, correspondence test with 1258 paired applications	Ethnic hierarchies among minority groups, ethnic-prominence-hypothesis	High skilled job (dentist) and intermediate skill level, female- and male-typed jobs	Austrian, Polish, Russian/Finnish men/women	Discrimination_*Polish male dentists*_ > discrimination_*Austrian male dentists*_; hiring probability: Austrian applicant = native Finnish applicant; discrimination_*Russian applicants*_ > discrimination_*Finnish applicants*_ when applying for semi-skilled gender-congruent jobs; discrimination_*Russian men*_ > discrimination_*Russian women*_ when applying for gender-incongruent jobs
Veit and Thijsen, 2019 Germany, Netherlands, Norway, Spain, United Kingdom	Correspondence test with 5954 unpaired applications	Taste-based and statistical discrimination	Intermediate skill level	Men/women born either in a European country or in a Middle Eastern/African country or born in the respective domestic country to minority group parents	Discrimination_*foreign–born applicants of African*__ or Middle–Eastern origin_ > discrimination_*foreign–born applicants*__ from other European countries_ and (in Spain and the Netherlands) _*domestic–born minorities*_
Weichselbaumer, 2016 Austria	Correspondence test with paired 2142 applications	Relevance of migration background	Female-/male-dominated jobs, intermediate skill level (office/hotel)	Men/women with migration background in Serbia, Turkey, China and Nigeria	Discrimination against all applicants with migration background (strongest against people of African origin)

### Double Jeopardy

The idea of a double jeopardy was first developed to describe the situation of Black women in the United States experiencing sexism from other Black men and racism from White men and women (King, 1975). It was expanded to point to other minority women as well. For example, when considering their sexual orientation, lesbians are part of two outgroups. Likewise, Black women, and women of color (e.g., Latina or Asian women) belong to two outgroups and should thus experience more discrimination than a person belonging to just one outgroup, like minority men, or majority women (Sidanius et al., 2000). Generally speaking, the double jeopardy hypothesis states that people belonging to multiple subordinate categories experience more discrimination than people who belong to just one subordinate social group. It thus makes contradictory predictions when compared to the SMTH. Within the double jeopardy approach, one can distinguish an interactive model assuming that the intertwined identities of multiple subordinated group members lead to a distinctive, integrated new identity (Settles, 2006). In contrast, the additive model examines discrimination primarily on the basis of racism and sexism (or other intersecting subordinate group memberships) and argues that these discriminations add up (Almquist, 1975).

When comparing labor market discrimination against people from Latin America in Spain and the United States, Yemane and Fernández-Reino (2019) noticed more discrimination against Latinx people in the United States. They attributed this to less cultural and linguistic proximity Latinx men and women share with United States-Americans compared to the inhabitants of Spain, the former mother country of many Latin American nations. Latino men and women in the United States are linked to illegal migration and are stereotyped as hypersexual machos and unskilled women (Yemane and Fernández-Reino, 2019). In Spain, Latino men and women are the most likable group of immigrants compared with other nations of origin. When looking at the consequences of being perceived as a Latinx person, Yemane and Fernández-Reino (2019) showed that ethnic stereotypes can be mitigated by additional individualizing information on communion. For example, stating that the applicant is seen as a warm, down-to-earth person by their friends and colleagues caused a significantly higher callback rate for Latinx men and women in the United States, but only for Latina women in Spain. This was especially pronounced in the absence of information regarding competence. All in all, Latino men experienced more discrimination than Latina women in the United States, whereas Latina women experienced more discrimination than Latino men in Spain, where Latino men were treated just like native-born Spanish men. Put differently, one could summarize that in the United States the ethnic prominence hypothesis seemed to explain the findings, whereas in Spain one could speak of a double jeopardy for Latina women. With regard to the skill level necessary, no clear pattern emerged in this study where applications were sent to jobs requiring low, medium, and high skill levels. Only when Latina women applied for high-skilled vacancies in Spain, they experienced discrimination compared to Latino men. Further evidence for the double jeopardy hypothesis is provided by the (above mentioned) studies in the Netherlands and Belgium by Derous and Ryan (2012) and Derous and Pepermans (2019). They showed that Arab women with a high ethnic identification applying for intermediate-skilled gender-neutral jobs experienced a double jeopardy as did Arab women when applying for a high-skilled manager position possibly due to the fact that high-status jobs are generally more gender-segregated, making applicants’ gender more salient. In Germany, women with a Turkish-sounding name who wore a headscarf were even more discriminated against than Turkish women without a headscarf (Weichselbaumer, 2020). Both were discriminated against compared to a woman with a German name. It is noteworthy that the photo used was always the same, only the name added differed and whether the applicant wore a headscarf or not. This procedure excludes the explanation that different callback rates are due to differences in likeability regarding various photos, but it does not meet the requirements for broad stimulus sampling (Wells and Windschitl, 1999). Thus, the generalizability of the finding is somewhat limited. The effect found in this study with one photo was more pronounced for jobs with a higher status than a lower status but consistent for different sectors and varying company sizes.

In sum, four studies (see Table 5 for an overview) thus point toward a double jeopardy for women based on their ethnic or religious, and gender identity, because they belonged to a racial minority and were identified as women. The findings seem more robust than the results on the SMTH detailed above. However, there are also many, very specific boundary conditions in these studies comparable to those that played a role regarding the ethnic prominence hypothesis. One of the studies (Derous and Ryan, 2012) did find a substantive effect in favor of the double jeopardy approach, yet it was non-significant. Its general applicability for different job contexts and identities can thus be doubted. Once again, it becomes clear that more research with different groups is generally needed. Contextual factors, like the required skill level for the job in question and its gender-typicality, must also be considered. In the three studies on a double jeopardy presented here only ethnic minority women experienced discrimination when applying for medium or high-skilled jobs. But does this translate to sexual minority women as well? We identify this as a pressing Research Need 2.

**TABLE 5:** Overview of European social psychological studies on labor market discrimination based on the double jeopardy theory.

Study	Sample	Theoretical framework	Job context	Social identities considered	Results
Derous and Ryan, 2012 Netherlands	Correspondence test: 600 paired (4 résumés each) applications	Ethnic prominence hypothesis, subordinate male target hypothesis	Intermediate skill level, gender-neutral jobs	Women/men with Arab names with activism in Dutch/Arab Youth organization	Discrimination_*Arab men*_ > discrimination_*Arab women*_ when applying for gender-neutral jobs requiring intermediate qualifications but only if they indicated activism in a Dutch and not an Arab Youth association; rejection_*Arab women*_ > rejection_*Arab men*_ when both highly identified as Arab
Derous and Pepermans, 2019 Belgium	Experiment: 214 Belgian recruiters	Double jeopardy hypothesis, ethnic-prominence-hypothesis	Low/high cognitive demand (clerk/manager)	Maghrebian/Arab men/women, high/low socioeconomic status of the Dutch applicant	No discrimination against Maghrebian/Arab women applying for low-cognitive demanding jobs in Belgium; when not considering job-context there was no difference in discrimination against Maghrebian/Arab men and women
Weichselbaumer, 2020 Germany	Correspondence test: 1474 unpaired applications	Intersectionality and head-scarf as stigma	Female-typed jobs, low/high status: office workers, small/international firm	Turkish woman with/without head-scarf and German woman	Discrimination against Turkish women with and without head-scarf, especially against Turkish women with head-scarf applying for high-status jobs
Yemane and Fernández-Reino, 2019 United States, Spain	Correspondence test: 1547 unpaired applications in Spain and 804 unpaired applications in the United States	Intersectionality, influence of competence and warmth signaled	Low/intermediate/high skill level, female-/male-typed jobs	Latino men/women	Discrimination against Latino men United States > Spain; strong discrimination against Latina women (but not Latino men) applying for higher skilled (and not typically female) jobs in Spain

Further, these results contrast with the finding by Arai et al. (2015) presented in the section on the SMTH that Arab women, no matter the amount of work experience they had, fared always better than their male counterparts when applying for both male- and female-typed jobs requiring medium and high skill levels. The authors attribute this to the fact that the Arab women were disconfirming the group stereotype of domestic wives associated with them. If this is a valid explanatory principle we should observe the same pattern regarding the three studies presented in this section as well. Thus, Research Need 3 is to shed light on the relevance of the stereotype content for different groups in explaining (no) discrimination.

The final concept presented here, intersectional invisibility, is the most comprehensive one, aiming to integrate the three above-mentioned perspectives and offering a way out of the score-keeping approach, which implicitly underlies the theories presented so far. Comparing and offsetting different forms of discrimination do not do justice to the complexity of discrimination or to people’s experiences of it. Intersectional invisibility accounts for the fact that belonging to a social group that does not constitute the norm can go along with both advantages and disadvantages depending on the specific combinations of group memberships.

### Intersectional Invisibility

The notion of intersectional invisibility brings to light that people who belong to multiple subordinate social groups are not prototypical of any one of those groups and may thus be rendered invisible not being recognized as members of the various groups they belong to (Crenshaw, 1989; Purdie-Vaughns and Eibach, 2008). This reasoning is based on norm theory backed up by empirical research on gender and sexual orientation. Category norms referring to the most typical exemplars of a category define what falls inside this category and what is perceived as abnormal for an exemplar of the respective category constituting the difference that needs to be explained (Hegarty and Pratto, 2004). Subsequently, stereotypes influence whether the explanation of this difference is based on enduring qualities of a group, i.e., essentialist, or rather on changeable circumstances, i.e., reconstructive (Hegarty and Pratto, 2004). Stereotype-consistent differences evoke more essentialist explanations, whereas stereotype-inconsistent differences evoke more reconstructive explanations (Hegarty and Pratto, 2004). Since the majority of social category norms are androcentric, women are most often seen as the deviating group whose behavior and attitudes need explanation compared to men (Hegarty and Pratto, 2004). Graphing the order of the sexes, this also affects perceptions of sexual orientation in such a way that the category of homosexual persons perceived in itself as a deviation from the heterosexual norm is rather seen as male/gay instead of female/lesbian (Hegarty and Pratto, 2004). This leads to an asymmetric explanation for group differences focusing on the group conceived as deviating from the norm (Hegarty and Bruckmüller, 2013). Regarding sexual orientation this pattern is called heterocentrism.

A second, sociological, tenet on which intersectional invisibility builds is ethnocentrism. It refers to the view that “one’s own group is the center of everything, and all others are scaled and rated with reference to it” (Sumner, 1906, p. 13). Social groups thus set their norms as a universally valid standard, while they judge other groups’ norms as invalid deviations. In multi-ethnic societies, it is the dominant social group that sets its norms as the standard and extends them to other groups. As has been shown, historically, White people formed the dominant social group in different societies that emerged as the West by distinguishing itself from the rest of the world. That’s why whiteness is generally the societal norm in Western nations (Bonilla-Silva, 2007; Arndt, 2017).

With androcentrism, heterocentrism, and ethnocentrism thus being three dominant ideologies, people who do not fit the respective prototypes of White heterosexual men are sort of falling through those categories. This invisibility can come with advantages as well as disadvantages (Purdie-Vaughns and Eibach, 2008).

As some examples for this intersectional invisibility of people at differing intersections of gender, race, ability, and sexual orientation in Germany, one can point to the often overlooked and actively concealed history of queer Jewish Holocaust survivors (Hájková, 2018), thus integrating once more the historical background into this review; to the role of Black lesbians in the women’s movement (Oguntoye et al., 2007); or the silence around the meaning of gender for people with mental disabilities and their struggle for asserting their reproductive and even physical rights: As one example, it is generally considered important to separate toilets by gender, but as soon as a person is in a wheelchair, this is perceived as totally superfluous (Langner, 2010).

Being under- or misrepresented makes it difficult for members of multiple subordinate groups to be listened to and to encounter an understanding of their experiences and realities of life. Here, the intersectional invisibility approach builds on the finding of social-identity theory that when group membership is salient, non-prototypical members of a group are less likely to be in leadership positions exerting social influence on other group members compared to prototypical members of the same group (Hogg, 2016).

Still, being invisible as a person with intersecting subordinate identities can come with the advantage of experiencing less direct discrimination (Purdie-Vaughns and Eibach, 2008). This allows a reinterpretation of the findings of the SMTH based on the non-prototypicality of women belonging to yet another subordinate group: Due to androcentrism, men belonging to another subordinate group are perceived as being more prototypical than women and therefore experience more often active discrimination (Purdie-Vaughns and Eibach, 2008).

Another example of an advantage due to intersectional invisibility can be found in a field experiment in six European countries. The comparative study on labor market discrimination was conducted in Germany, Norway, United Kingdom, Spain, and the Netherlands (Di Stasio and Larsen, 2020). A concrete job context was given, with jobs requiring either an apprenticeship or a university degree. Members of racialized minorities were discriminated against more than foreign White applicants. White female applicants were preferred regardless of a migrant background when applying for congruent gender-typed jobs. In typically female professions, racialized women were discriminated against more than racialized men. This finding can be interpreted in terms of the intersectional invisibility that minority men in female-dominated professions experience due to their non-prototypical race and gender rendering them less obvious targets of discrimination. In contrast, Black and Arab men were most discriminated against in typically male professions possibly due to the role-congruity of their gender and job context which made their perceived threatening masculinity more salient. Black women, however, experienced stronger discrimination than White foreign and Asian women regardless of the gender distribution in the industry to which they were applying. Only with an increasing proportion of men in the respective occupational field were Black women slightly less discriminated against. We propose that this could be due to the stereotype of Black women as being more masculine and dominant than White women (Rosette et al., 2016; Ball, unpublished) and thus more apt for typically male jobs. When a working context was given in a German study (Niedlich and Steffens, 2017) the results were different: Here, it was varied whether the job was typically male (police officer) or female (primary school teacher) with both jobs requiring higher education. Applying for the gender-atypical job, neither sexual- and ethnic-minority women and men, nor German gay/lesbian applicants received lower hireability, agency, and communion ratings. Thus, in this study, the specific combination of social identities in interaction with the job context only led to advantages for minority group members.

Even though not including race, other findings regarding the intersections of gender and sexual orientation can inform the intersectional invisibility hypothesis in the context of European racism as well. German lesbians seen as deviating regarding both masculine gender and heterosexual orientation were likewise evaluated more positively as shown by Niedlich and Steffens (2015): In hypothetical job interviews, lesbians were rated as more competent than heterosexual women if they showed typically female behavior (moving town because of their partner’s job). Both German lesbians and gay men were described as more competent and socially skilled than heterosexual applicants in another study (Niedlich and Steffens, 2015). Yet, there were no advantages regarding the likelihood of being hired for a high- or low-qualified management position due to the ascribed higher competence and communion. A similar finding for White gay compared to White straight men is reported by Steffens et al. (2018). Heterosexual men were seen as more gender-typed than were gay men, and gay men were seen as more gay-stereotypical than were heterosexual men. No discrimination in the likelihood of being hired for a job that requires both agency and communion was found. When the job context was varied in a second experiment, gay men appeared more suitable for traditionally female jobs and vice versa for heterosexual men.

In summary, it can be seen that the intersection of gender and sexual orientation leads to different impressions of applicants’ agentic and communal traits. Furthermore, the specific job context plays also a role in assessing applicants’ suitability for the respective position.

All in all, the findings of all these studies (see Table 6) can be seen as examples of the “distinctive mixture of advantages and disadvantages that people with intersecting identities” experience (Purdie-Vaughns and Eibach, 2008, p. 380). Here, the advantages and disadvantages were either at balance or the advantages that minority applicants experienced were even more pronounced.

**TABLE 6:** Overview of European social psychological studies on labor market discrimination based on the theory of intersectional invisibility.

Study	Sample	Theoretical framework	Job context	Social identities considered	Results
Niedlich and Steffens, 2015 Germany^1^	Experiment: 266 people (undergraduate students and convenience sample)	Lack-of-fit-model, backlash-effect, transgression of gender-roles	High status, female-/male-typed job	Heterosexual/gay or lesbian men/women, low/high qualification	Competence, social skills_*lesbian/gay applicants*_ > competence, social skills_*heterosexual applicants*_, more positive attitude toward gays/lesbians: hireability_*gay/lesbian applicants*_ > hireability_*heterosexual applicants*_
Niedlich et al., 2015 Germany^2^	Experiment 1: 118 young heterosexual men/women, experiment 2: 158 undergraduate students	Lack-of-fit-model, transgression of gender-roles	Gender-neutral job	Heterosexual/gay or lesbian women/men	Competence_*lesbians*_ > competence_*heterosexual women*_ (when showing typically female behavior) competence and masculinity_*heterosexual women*_ < competence and masculinity_*lesbians*_, high motivation to appear non-prejudiced in second sample: no effect
Niedlich and Steffens, 2017 Germany	Experiment 1: 433 raters, experiment 2: 379 raters, experiment 3: 275 raters	Intersectionality	High skill level, female-typed and gender-neutral job	Turkish/German heterosexual/gay or lesbian men/women	Lesbian/Turkish women, gay/Turkish men: no discrimination in gender-atypical job neither for German gays/lesbians
Steffens et al., 2018 Germany	Experiment 1: 273 participants, experiment 2: 32 female students	Lack-of-fit-model	Gender-neutral job, male-/female-typed jobs with intermediate/high skill level	Heterosexual/gay men	Gender-typed_*heterosexual men*_ > gender-typed_*gay men*_ gay-stereotypical_*gay men*_ > gay-stereotypical_*heterosexual men*_, warmth_*gay men*_ > warmth_*heterosexual men*_, hireability for a job requiring agency and communion: gay men = heterosexual men
Strinić et al., 2020 Sweden	Experiment: 133 recruiters and employees with work experience	Warmth and competence ratings, integration of Stereotype Content Model with intersectionality	No job context	Arab/Swedish name, gender, sexual orientation, age with combinations of ethnicity, gender, age and ethnicity, gender and sexual orientation	Warmth_*young Arab women*_ > warmth_*older Arab women*_ (contrary to Swedish women); Swedish lesbians: competence > warmth; Swedish gay men: competence < warmth; Arab gay men: competence = heterosexual Arab men, warmth > heterosexual Arab men
Di Stasio and Larsen, 2020 Germany, Netherlands, Norway, Spain, United Kingdom	Correspondence test: 19000	Intersectionality	Low/high skill level, low/high status, male- and female-typed jobs	Whites without migration background, migrants being white/black or having an Asian/Middle Eastern and North African migration background	Gender-congruent jobs: White women (with/without migration background) preferred, female-dominated profession: discrimination_*minority women*_ > discrimination_*minority men*_, Black/Arab men most discriminated against in male-dominated professions, discrimination_*Black women*_ > discrimination_*white foreign/Asian women*_

*^1^Rater characteristics: prejudiced attitudes toward gay men and lesbians.*

*^2^Rater characteristics: motivation to appear non-prejudiced.*

In future studies, the interesting question to explore further is whether this is true for non-White gender and sexual-orientation minorities as well. Recent results from our lab (Ball, unpublished) described in more detail above under Section “Empirical Pieces of the Puzzle” showed that, descriptively, Black lesbians were rated as more agentic and also more competent than White and Black heterosexual women, and that Black lesbians were rated as similarly agentic as White men. Based on these findings we expect an interaction effect of sexual orientation and skin color (Rosette and Livingston, 2012) when replicating the experiment by Niedlich et al. (2015) on the evaluation of heterosexual and lesbian applicants. We expect that Black lesbians will appear similarly agentic as White heterosexual men and even more agentic than both Black heterosexual women and White lesbians when applying for a job and showing typically female behavior.

Since Black gay men’s sexual orientation leads to a de-racialization (Petsko and Bodenhausen, 2019; Strinić et al., 2020), we expect to find that Black gay men will be ascribed more communion than Black heterosexual men and similar communion as white heterosexual men when replicating Petsko and Bodenhausen’s (2019) experiment on agency and communion assessments of straight and gay men within a European context. A study in Sweden (Strinić et al., 2020) examining the two fundamental dimensions of person perception, agency and communion, as two highly relevant categories in selection decisions for native Swedish and Arab people with differing combinations of race, gender, and sexual orientation, respectively, race, gender, and age, presented some hints that Arab gay men were de-racialized. They were perceived as possessing a similar level of competence as heterosexual Arab men, but higher warmth. However, as Research Need 4, clarification is needed whether this is also explained by an ascribed higher SES and by more femininity (Petsko and Bodenhausen, 2019).

Moreover, only one of the four presented studies that took an intersectional approach in examining racial discrimination at the European labor market did include sexual minorities. Thus, the predictions and postulated mechanisms of intersectional invisibility need to be explored in more detail to shed light on the role of the respective stereotype-content as agency and communion assessments change with applicants’ sexual orientation (Niedlich et al., 2015; Wilson et al., 2017; Steffens et al., 2018; Strinić et al., 2020). Hereby it is important to keep in mind the differential racialization (Delgado and Stefancic, 2001) and different stereotypes connected with social groups which should interact in varying ways with other relevant situational features like the job context or the required skill-level as well as with other social group memberships. Regarding the influence of the job status (connected to the required skill-level), we have indicated that it clearly needs to be taken into account as well.

## Discussion

### Missing Groups and Intersections

To sum up, a variety of different explanatory approaches have been brought forward to describe the complex process of impression formation and evaluation of people with intersecting social identities. Still, more research is needed that looks at different groups and their intersections in different contexts in order to establish conditions under which one or the other of these explanatory approaches is supported. With Research Needs 1–4 we identified the most promising questions to pursue further: A clarification is needed which role the job context (high/low status, typically female/male job) plays in explaining (no) discrimination; what pattern of discrimination or preference is found when sexual orientation comes into play; the role of stereotype content keeping in mind the differential racialization of marginalized groups (Delgado and Stefancic, 2001), gendered racism (Galinsky et al., 2013; Hall et al., 2015), and, on a more general level, the stereotype content model (Fiske et al., 2002) so that a broadening of the scope of groups and forms of racism considered (e.g., racism against Rom:nja and Sinti:zze, Anti-Asian racism, Antisemitism) seems necessary; finally, we pointed toward the open question of whether de-racialization processes mediated via higher ascribed SES matter in Europe as well. The category of SES is missing in almost all of the research on labor market discrimination. We consider it a promising Research Need 5 to take class differences into account, in particular, because a low level of education and poor language skills are often used as a legitimation for discrimination (Gomolla and Radtke, 2009; Hansen et al., 2013; Schmaus, 2018). Only a few studies have looked at class-based discrimination from an intersectional perspective. On the one hand, in a correspondence test in Denmark (Dahl and Krog, 2018) SES of the majority-group candidates was manipulated via the names given to the Danish applicants. Racial discrimination against applicants with Middle-Eastern names was not reduced when their Danish counterpart had a low SES, rather, the discrimination of the applicant with a Middle Eastern-sounding name was even higher in this case. The authors conclude thus that only racial discrimination was at work, not SES-based discrimination. Similarly, Derous et al. (2012) did not find an effect of class when manipulating place of residence and type of school attended in job applications. In contrast, Jackson (2009) found in the United Kingdom that people signaling high SES (manipulated via university/school attended, hobbies, name) had a small advantage of receiving callbacks regarding their applications for middle-class jobs. Having an elite name accounted for most of this advantage. The above-mentioned study on the de-racialization of gay Black men (Petsko and Bodenhausen, 2019) also points to a linkage between SES and racialization but needs to be replicated in the European context. In their United States-based study, Black, White, Asian, and Hispanic men were all de-racialized when described as gay. But only Black men described as having a low socioeconomic background were attributed a higher SES and consequently were described as Whiter when they were presented as gay rather than heterosexual. Our own research (Ball, unpublished) hints toward subtyping in the perception of Black people in Germany. Their ascribed foreignness seemed to be connected to seeing them either as African Americans or as Africans. Since only the former subtype may be associated with a higher SES, we expect the effect found by Petsko and Bodenhausen (2019) to apply only here. By implication, research in Germany (and probably the whole of Europe) needs to take into account whether a Black stimulus person is interpreted as African American or African, and this could depend on experimental condition (e.g., high-status job = African American, low-status job = African).

Finally, age-based discrimination in combination with other social categories is hardly researched, either. This presents Research Need 6 as only two studies in Sweden (Carlsson and Eriksson, 2019; Strinić et al., 2020) looked at this form of discrimination. In a country where the participation of older people, mostly women, in the labor force is high, age-based discrimination was found to be especially pronounced for women in the lower section of the age interval of 35 to 70. This was due to a higher callback rate for the younger women compared to men of the same age. The finding held for low- and medium-skilled jobs in both gender-neutral and male- and female-dominated areas. The authors of the first study (Carlsson and Eriksson, 2019) conducted a survey with recruiters in the wake of the correspondence. Based on the results they presented hints that employers discriminate against older people – or applicants who are no longer that young – because they ascribe them a lack of flexibility, learning capacity, and ambition. The above-mentioned survey by Strinić et al. (2020) did also consider age besides race, gender, and sexual orientation. Contrary to Swedish women, Arab women were perceived as warmer when of young age rather than being 55 years old. In contrast, Swedish women were rated as warmer the older they were. All in all, race mattered more when it came to differences regarding competence, and gender and sexual orientation mattered more when it came to differences regarding warmth. Age did not play a role as important as race and gender and it was only relevant for female applicants, similar to Carlsson and Eriksson’s (2019) results.

Once again, this points to the need to take into account specific stereotype content at identity intersections. Additionally, we want to sketch the potential contribution of the social-identity approach to understanding intersectional discrimination.

### Enriching Intersectional Research With the Social-Identity Approach

As we have argued and reviewed in Tables 3–6, each of the different intersectional predictions has received some empirical support, but is also faced with contradictory findings. This research-stage suggests that moderating factors determine who is discriminated, or not, under different conditions. The best established theoretical approach that considers the intergroup-structure in the whole context, a European tradition in social psychology, is the social-identity approach. It encompasses social-identity theory and self-categorization theory (Tajfel and Turner, 1979; Turner et al., 1987). The main tenet of social-identity theory is that people’s identities are based on social groups they belong to. This categorization into ‘us’ versus ‘them’ can be a basis for conflictual intergroup relations. Comparisons with other groups are used to maintain or obtain positive social identities. A review of this rich approach – revised and supported in hundreds of studies – is beyond the scope of the present paper (see Brown, 2000, for a critical review). But we want to sketch several of its predictions and findings that could inform research on intersectional racial discrimination.

#### Flexibility of Social Categorization

Each individual belongs to multiple social groups at once. Therefore, individuals who are categorized as ingroup members in one context can be categorized as outgroup members in another. For example, based on their appearance, people were regarded as ingroup members, but when they started to speak with an accent, they became outgroup members (Rakić et al., 2011). The topic of discussion may also determine which category is applied (e.g., age vs. gender, Klauer et al., 2003). This flexibility of social categorization suggests that general intersectional hypotheses such as the SMTH should not apply under all conditions, but only under specific ones (i.e., at least both race and gender should be relevant in the context-at-hand). As one example, if people are categorized as members of one sports team irrespective of skin-color (Kurzban et al., 2001), it is obvious that there is no discrimination against Black men.

#### Nested Social Categories

Not only are social categories crossed as indicated in the previous paragraph, but they are also nested within each other. An outgroup member on a lower level of inclusion can thus be an ingroup member on a higher level. A simple manipulation such as a questionnaire on ‘being a Manchester United fan’ versus ‘being a football fan’ can determine behavior toward an alleged Liverpool fan (Levine et al., 2005). As an example more pertinent to the present topic, Black and White people could all be included in the common ingroup of United States-Americans (cf. Gaertner et al., 2000). Whereas inclusion in a common ingroup can be a basis for positive attitudes and behavior, the shared superordinate group can also provoke contest pertaining to the question who defines best the superordinate group (see Wenzel et al., 2016): For example, who are the ‘real’ Europeans? (Waldzus et al., 2005; Bianchi et al., 2010). Moreover, according to the black sheep effect (e.g., Reese et al., 2013), sometimes ingroup members are judged even more harshly than outgroup members. The fact that social categories can be used at different levels of inclusion has many implications for intersectional theories. For example, the black sheep effect could be an explanation for the discrimination of prototypes as compared to targets with multiple minority status: Outgroup members could be exempt from the norms applied to ingroup members; such that an autochthonous woman should know a certain norm, a ‘foreigner’ may not. As another example, an absence of discrimination based on targets’ social group membership could occur if these social groups are perceived as irrelevant in the context-at-hand and all targets are similarly categorized as superordinate-group members (e.g., as leaders within a company).

#### Threat

A third way in which the social-identity approach may inform findings on intersectional discrimination, in addition to the flexibility of the type and level of social categorization, is that various types of threat have been discussed within the scope of this theory (Branscombe et al., 1999). For instance, too much perceived similarity between ingroup and outgroup can provoke distinctiveness threat (Maass et al., 2003). As this implies, a further important contextual factor determining behavior toward outgroups is whether intergroup relations are perceived as stable or whether participants feel threatened by the outgroup or its members.

Taken together, we argue that a social-identity lens can fruitfully be applied to the somewhat muddled and conflicting results that the subordinate male target hypothesis, the double jeopardy approach, the ethnic prominence hypothesis and the intersectional invisibility theory provide. When examining intersectional discrimination with a focus on racism, social-identity theory and self-categorization theory offer promising explanatory approaches to classify the inconsistent findings on intersectional discrimination.

## Conclusion

Since intersections cannot be studied without including concrete stereotypes as the stereotype content model shows (Fiske et al., 2002), it is highly unlikely that either the subordinate male target hypothesis, the ethnic prominence hypothesis, the double jeopardy approach, or the theory of intersectional invisibility alone will fit all groups. When complemented by insights from the social-identity approach (Tajfel and Turner, 1979; Turner et al., 1987) crossed categorizations, nested social categories, and identity-threats can help explain which of the four intersectional approaches is the most adequate one for explaining principles and mechanisms of discrimination.

Still, an intersectional perspective is important to do justice to the complex reality and can adequately describe the interaction of categories of difference. Thus, “[r]ather than asking who is more disadvantaged, we should instead ask how the forms of oppression that people with intersecting disadvantaged identities experience differ from the forms of oppression that people with a single disadvantaged identity experience” (Purdie-Vaughns and Eibach, 2008, p. 380). At the same time, it is not yet fully understood what role a concrete job-context plays, if de-racialization processes are based on ascriptions of higher SES to individuals, whether stereotype contents for different marginalized groups can explain partly conflicting results, and what the picture of (no) discrimination looks like when other relevant social categories, like sexual orientation, disability, or age, are considered.

These questions need to be addressed to further our understanding of the complex reality of intersectional discrimination in Europe.

## Data Availability Statement

The original contributions presented in the study are included in the article/supplementary material, further inquiries can be directed to the corresponding author/s.

## Ethics Statement

Ethical review and approval was not required for the study on human participants in accordance with the local legislation and institutional requirements. The patients/participants provided their written informed consent to participate in this study.

## Author Contributions

EB drafted the manuscript. MS, CN, and EB revised it several times. All authors approved the final version.

## Conflict of Interest

The authors declare that the research was conducted in the absence of any commercial or financial relationships that could be construed as a potential conflict of interest.

## Publisher’s Note

All claims expressed in this article are solely those of the authors and do not necessarily represent those of their affiliated organizations, or those of the publisher, the editors and the reviewers. Any product that may be evaluated in this article, or claim that may be made by its manufacturer, is not guaranteed or endorsed by the publisher.

## Footnotes

1 Ball, E. (2021). *What Does it Mean to be Black in Germany? Evaluation of the Stereotype Content Towards Black People in Germany* [unpublished data]. Landau: University of Koblenz-Landau.
